# Bis(μ-2,2′-disulfanediyldibenzoato)bis­[aqua­(2,2′-bipyridine)­nickel(II)]

**DOI:** 10.1107/S1600536810045824

**Published:** 2010-11-13

**Authors:** Zhengming Liu, Botao Qu, Jianghua Yu, Limin Yuan, Wenlong Liu

**Affiliations:** a Testing Center, Yangzhou Universitry, Yangzhou, 225002, People’s Republic of China; b College of Chemistry and Chemical Engineering, Yangzhou Universitry, Yangzhou, 225002, People’s Republic of China

## Abstract

In the centrosymmetric title complex, [Ni_2_(C_14_H_8_O_4_S_2_)_2_(C_10_H_8_N_2_)_2_(H_2_O)_2_], the Ni^II^ atom is coordinated by two N atoms from one 2,2′-bipyridine ligand, three carboxyl­ate O atoms (one bidentate and one monodentate) from two different disulfanediyldibenzoate ligands and one O atom from a coordinated water mol­ecule in an octa­hedral coordination geometry. The disulfanediyldibenzo­ate dianion bridges two Ni^II ^atoms. Adjacent mol­ecules are linked through the coordinated water mol­ecules, forming a O—H⋯O hydrogen-bonded chain running along the *a* axis.

## Related literature

For complexes of 2,2′-disulfanediyldibenzoic acid, see: Feng *et al.* (2009 ▶); Humphrey *et al.* (2004 ▶); Li *et al.* (2007 ▶); Murugavel *et al.* (2001 ▶); Zhou *et al.* (2009 ▶).
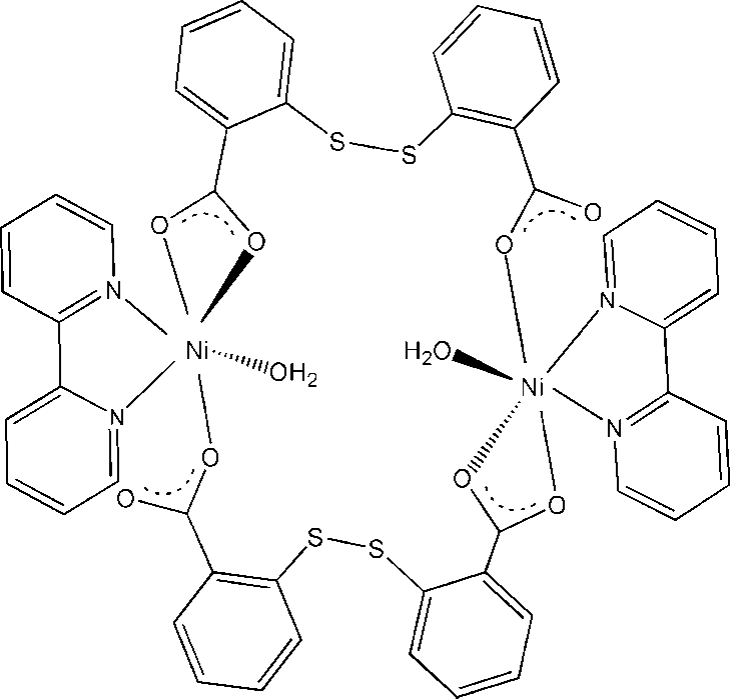


## Experimental

### 

#### Crystal data

[Ni_2_(C_14_H_8_O_4_S_2_)_2_(C_10_H_8_N_2_)_2_(H_2_O)_2_]
*M* *_r_* = 1074.47
Monoclinic, 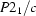

*a* = 13.498 (4) Å
*b* = 16.769 (5) Å
*c* = 10.238 (3) Å
β = 93.196 (4)°
*V* = 2313.7 (12) Å^3^
*Z* = 2
Mo *K*α radiation
μ = 1.06 mm^−1^
*T* = 296 K
0.35 × 0.33 × 0.28 mm

#### Data collection

Bruker SMART APEX CCD diffractometer
Absorption correction: multi-scan (*SADABS*; Sheldrick, 2004 ▶) *T*_min_ = 0.708, *T*_max_ = 0.756
19861 measured reflections
5302 independent reflections
4428 reflections with *I* > 2σ(*I*)
*R*_int_ = 0.034

#### Refinement

*R*[*F* ^2^ > 2σ(*F* ^2^)] = 0.029
*wR*(*F* ^2^) = 0.080
*S* = 1.06
5302 reflections
307 parameters
3 restraints
H atoms treated by a mixture of independent and constrained refinement
Δρ_max_ = 0.34 e Å^−3^
Δρ_min_ = −0.26 e Å^−3^

### 

Data collection: *SMART* (Bruker, 2002) ▶; cell refinement: *SAINT-Plus* (Bruker, 2003 ▶); data reduction: *SAINT-Plus*; program(s) used to solve structure: *SHELXTL* (Sheldrick, 2008 ▶); program(s) used to refine structure: *SHELXTL*; molecular graphics: *SHELXTL* and *DIAMOND* (Brandenburg, 2006 ▶); software used to prepare material for publication: *publCIF* (Westrip, 2010 ▶).

## Supplementary Material

Crystal structure: contains datablocks I, global. DOI: 10.1107/S1600536810045824/ng5064sup1.cif

Structure factors: contains datablocks I. DOI: 10.1107/S1600536810045824/ng5064Isup2.hkl

Additional supplementary materials:  crystallographic information; 3D view; checkCIF report

## Figures and Tables

**Table 1:** Hydrogen-bond geometry (Å, °)

*D*—H⋯*A*	*D*—H	H⋯*A*	*D*⋯*A*	*D*—H⋯*A*
O5—H5*B*⋯O2	0.85 (1)	1.82 (1)	2.646 (2)	162 (2)
O5—H5*A*⋯O4^i^	0.84 (1)	1.89 (1)	2.7187 (18)	169 (2)

Symmetry code: (i) 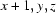.

**Fig. 1.**
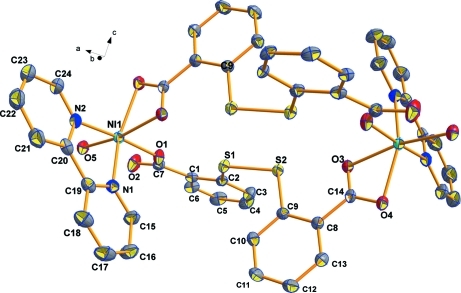
The molecular structure of the title compound, showing the atom-labelling scheme and displacement ellipsoids drawn at the 35% probability level. Hydrogen atoms and are omitted for clarity.

**Fig. 2.**
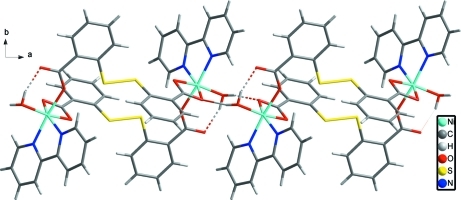
View of the one-dimensional hydrogen-bonded chain of the title compound running along the a axis.

## supplementary crystallographic information

### Comment

The flexible 2,2'-disulfanediyldibenzoic acid, a multifunctional ligand containing both carboxylic and thio groups, can potentially afford various coordination modes and diverse coordination architectures. Many complexes with this ligand show unique structural topologies and interesting properties (Murugavel *et al.*, 2001; Humphrey *et al.*, 2004; Li *et al.*, 2007; Zhou *et al.*, 2009; Feng *et al.*, 2009). In this work, we have used this ligand to react with a Ni^II^ salt in the presence of 2,2'-bipyridine as a chelating co-ligand, to obtain the title binuclear compound, Ni_2_(H_2_O)_2_(C_10_H_8_N_2_)_2_(C_14_H_8_O_4_S_2_)_2_.

The asymmetric unit of the title compound is composed of one Ni^II^ ion, one 2,2'-disulfanediyldibenzoate ligand, one 2,2'-bipyridine ligand and one coordinated water molecule (Fig. 1). The Ni^II^ center is six-coordinated, in a distorted octahedral geometry, by two N atoms from one 2,2'-bipyridine ligand, three carboxylate O atoms from two different disulfanediyldibenzoate ligands and one O atom from a coordinated water molecule Two disulfanediyldibenzoate dianions bridge two Ni^II ^ions about a center of inversion with its two carboxylate groups in bidentate chelating and monodentate modes, respectively, generating the title binuclear compound. The Ni···Ni distance bridged by two 2, 2'-disulfanediyldibenzoate is 10.061 (3) Å. Adjacent molecules are linked through both O5—H5A···O2 and O5—H5B···O4(*x* + 1, *y*, *z*) hydrogen bonds and lead to the formation of a one-dimensional hydrogen-bonded chain running along the *a* axis (Fig. 2).

### Experimental

A mixture of Ni(NO_3_)_2_.6H_2_O (21.0 mg, 0.1 mmol) with 2,2'-disulfanediyldibenzoic acid (30.6 mg, 0.1 mmol), 2,2'-bipyridine (15.6 mg, 0.1 mmol) and water (10 ml) was sealed in a 25 ml Teflon-lined stainless steel vessel, and heated at 383 K for 3 d. A number of green block crystals of (I) were obtained after cooling the solution to room temperature. The yield of (I) was *ca* 65%, based on 2, 2'-disulfanediyldibenzoic acid.

### Refinement

The water H atoms were located in a difference Fourier map with a distance restraint of O—H = 0.85 Å and *U*_iso_(H) =1.5*U*_eq_(O). The refinement of water H atoms were performed using 3 least-squares restraints by applying *DFIX* instructions of *SHELXTL*. All other H atoms were positioned geometrically and constrained as riding atoms, with C—H distances of 0.93 Å and *U*_iso_(H) set to 1.2_eq_(C) of the parent atom.

### Crystal data

**Table:**

[Ni_2_(C_14_H_8_O_4_S_2_)_2_(C_10_H_8_N_2_)_2_(H_2_O)_2_]	*F*(000) = 1104
*M**_r_* = 1074.47	*D*_x_ = 1.542 Mg m^−^^3^
Monoclinic, *P*2_1_/*c*	Mo *K*α radiation, λ = 0.71073 Å
Hall symbol: -P 2ybc	Cell parameters from 7893 reflections
*a* = 13.498 (4) Å	θ = 2.3–27.5°
*b* = 16.769 (5) Å	µ = 1.06 mm^−^^1^
*c* = 10.238 (3) Å	*T* = 296 K
β = 93.196 (4)°	Block, green
*V* = 2313.7 (12) Å^3^	0.35 × 0.33 × 0.28 mm
*Z* = 2	

### Data collection

**Table:**

Bruker SMART APEX CCD diffractometer	5302 independent reflections
Radiation source: fine-focus sealed tube	4428 reflections with *I* > 2σ(*I*)
graphite	*R*_int_ = 0.034
φ and ω scans	θ_max_ = 27.5°, θ_min_ = 1.5°
Absorption correction: multi-scan (*SADABS*; Sheldrick, 2004)	*h* = −17→17
*T*_min_ = 0.708, *T*_max_ = 0.756	*k* = −21→20
19861 measured reflections	*l* = −13→13

### Refinement

**Table:**

Refinement on *F*^2^	Primary atom site location: structure-invariant direct methods
Least-squares matrix: full	Secondary atom site location: difference Fourier map
*R*[*F*^2^ > 2σ(*F*^2^)] = 0.029	Hydrogen site location: inferred from neighbouring sites
*wR*(*F*^2^) = 0.080	H atoms treated by a mixture of independent and constrained refinement
*S* = 1.06	*w* = 1/[σ^2^(*F*_o_^2^) + (0.0369*P*)^2^ + 0.5543*P*] where *P* = (*F*_o_^2^ + 2*F*_c_^2^)/3
5302 reflections	(Δ/σ)_max_ = 0.001
307 parameters	Δρ_max_ = 0.34 e Å^−^^3^
3 restraints	Δρ_min_ = −0.26 e Å^−^^3^

### Special details

**Table:**

Geometry. All e.s.d.'s (except the e.s.d. in the dihedral angle between two l.s. planes) are estimated using the full covariance matrix. The cell e.s.d.'s are taken into account individually in the estimation of e.s.d.'s in distances, angles and torsion angles; correlations between e.s.d.'s in cell parameters are only used when they are defined by crystal symmetry. An approximate (isotropic) treatment of cell e.s.d.'s is used for estimating e.s.d.'s involving l.s. planes.
Refinement. Refinement of *F*^2^ against ALL reflections. The weighted *R*-factor *wR* and goodness of fit *S* are based on *F*^2^, conventional *R*-factors *R* are based on *F*, with *F* set to zero for negative *F*^2^. The threshold expression of *F*^2^ > σ(*F*^2^) is used only for calculating *R*-factors(gt) *etc*. and is not relevant to the choice of reflections for refinement. *R*-factors based on *F*^2^ are statistically about twice as large as those based on *F*, and *R*- factors based on ALL data will be even larger.

### Fractional atomic coordinates and isotropic or equivalent isotropic displacement parameters (Å^2^)

**Table:**

	*x*	*y*	*z*	*U*_iso_*/*U*_eq_	
Ni1	0.847531 (15)	0.055670 (13)	0.87112 (2)	0.035	
C1	0.71145 (13)	−0.16433 (10)	0.72485 (16)	0.0372 (4)	
C2	0.61332 (13)	−0.15067 (10)	0.75883 (16)	0.0361 (4)	
C3	0.54059 (16)	−0.20699 (12)	0.7226 (2)	0.0516 (5)	
H3	0.4753	−0.1986	0.7440	0.062*	
C4	0.56481 (19)	−0.27532 (13)	0.6549 (2)	0.0630 (6)	
H4	0.5157	−0.3124	0.6317	0.076*	
C5	0.66066 (19)	−0.28886 (13)	0.6217 (2)	0.0635 (6)	
H5	0.6763	−0.3347	0.5760	0.076*	
C6	0.73315 (17)	−0.23407 (12)	0.65659 (19)	0.0514 (5)	
H6	0.7980	−0.2435	0.6344	0.062*	
C7	0.79432 (13)	−0.10570 (11)	0.75377 (16)	0.0375 (4)	
C8	0.26979 (12)	0.00106 (10)	0.79602 (16)	0.0364 (4)	
C9	0.37029 (12)	−0.01665 (10)	0.77860 (15)	0.0352 (3)	
C10	0.41443 (15)	0.01522 (13)	0.67009 (18)	0.0493 (5)	
H10	0.4808	0.0044	0.6576	0.059*	
C11	0.36100 (17)	0.06275 (15)	0.5808 (2)	0.0609 (6)	
H11	0.3919	0.0836	0.5094	0.073*	
C12	0.26263 (17)	0.07935 (15)	0.5966 (2)	0.0631 (6)	
H12	0.2268	0.1108	0.5359	0.076*	
C13	0.21750 (15)	0.04884 (13)	0.70385 (19)	0.0509 (5)	
H13	0.1511	0.0603	0.7149	0.061*	
C14	0.21825 (12)	−0.02608 (10)	0.91345 (16)	0.0345 (3)	
O1	0.76966 (9)	−0.04232 (7)	0.81054 (13)	0.0430 (3)	
O2	0.87892 (10)	−0.12087 (10)	0.71985 (16)	0.0652 (4)	
O3	0.26151 (9)	−0.07316 (7)	0.99322 (11)	0.0361 (3)	
O4	0.13146 (8)	−0.00058 (8)	0.93571 (12)	0.0437 (3)	
S1	0.58588 (3)	−0.06272 (3)	0.85144 (4)	0.03707 (9)	
S2	0.44006 (3)	−0.07593 (3)	0.89692 (4)	0.03871 (10)	
C15	0.71731 (16)	0.10390 (13)	0.6351 (2)	0.0548 (5)	
H15	0.7039	0.0497	0.6255	0.066*	
C16	0.66890 (17)	0.15698 (15)	0.5504 (2)	0.0650 (6)	
H16	0.6246	0.1389	0.4841	0.078*	
C17	0.68751 (18)	0.23695 (15)	0.5661 (2)	0.0652 (6)	
H17	0.6560	0.2739	0.5103	0.078*	
C18	0.75337 (18)	0.26211 (13)	0.6655 (2)	0.0577 (5)	
H18	0.7656	0.3162	0.6782	0.069*	
C19	0.80122 (14)	0.20630 (11)	0.74626 (18)	0.0430 (4)	
C20	0.87518 (14)	0.22631 (11)	0.85377 (18)	0.0441 (4)	
C21	0.90507 (19)	0.30399 (14)	0.8850 (2)	0.0629 (6)	
H21	0.8783	0.3471	0.8380	0.075*	
C22	0.9751 (2)	0.31585 (16)	0.9869 (3)	0.0739 (7)	
H22	0.9951	0.3673	1.0099	0.089*	
C23	1.01454 (18)	0.25211 (16)	1.0536 (2)	0.0671 (6)	
H23	1.0621	0.2593	1.1219	0.081*	
C24	0.98269 (15)	0.17672 (14)	1.0180 (2)	0.0564 (5)	
H24	1.0101	0.1332	1.0631	0.068*	
N1	0.78256 (11)	0.12735 (9)	0.73016 (14)	0.0413 (3)	
N2	0.91367 (11)	0.16354 (9)	0.92081 (15)	0.0432 (3)	
O5	0.96680 (9)	0.01730 (9)	0.77302 (14)	0.0491 (3)	
H5A	1.0177 (12)	0.0183 (13)	0.824 (2)	0.074*	
H5B	0.9511 (17)	−0.0306 (7)	0.754 (2)	0.074*	

### Atomic displacement parameters (Å^2^)

**Table:**

	*U*^11^	*U*^22^	*U*^33^	*U*^12^	*U*^13^	*U*^23^
Ni1	0.026	0.039	0.040	−0.001	0.003	0.007
C1	0.0405 (9)	0.0373 (9)	0.0332 (8)	0.0051 (7)	−0.0024 (7)	0.0006 (7)
C2	0.0394 (9)	0.0346 (9)	0.0338 (8)	0.0004 (7)	−0.0025 (7)	0.0028 (6)
C3	0.0476 (11)	0.0493 (11)	0.0572 (11)	−0.0082 (9)	−0.0033 (9)	−0.0062 (9)
C4	0.0699 (15)	0.0470 (12)	0.0703 (14)	−0.0087 (11)	−0.0129 (12)	−0.0120 (10)
C5	0.0832 (17)	0.0434 (12)	0.0624 (13)	0.0081 (11)	−0.0098 (12)	−0.0160 (10)
C6	0.0570 (12)	0.0489 (11)	0.0476 (10)	0.0123 (10)	−0.0028 (9)	−0.0067 (9)
C7	0.0341 (9)	0.0441 (10)	0.0340 (8)	0.0070 (7)	0.0008 (7)	0.0009 (7)
C8	0.0318 (8)	0.0419 (9)	0.0356 (8)	−0.0026 (7)	0.0020 (7)	−0.0001 (7)
C9	0.0343 (8)	0.0380 (9)	0.0333 (8)	−0.0011 (7)	0.0009 (6)	−0.0010 (7)
C10	0.0398 (10)	0.0672 (13)	0.0416 (9)	0.0035 (9)	0.0100 (8)	0.0056 (9)
C11	0.0549 (13)	0.0874 (17)	0.0411 (10)	0.0048 (11)	0.0106 (9)	0.0210 (10)
C12	0.0552 (13)	0.0885 (17)	0.0455 (11)	0.0112 (12)	0.0017 (10)	0.0251 (11)
C13	0.0362 (10)	0.0692 (14)	0.0474 (10)	0.0067 (9)	0.0025 (8)	0.0131 (9)
C14	0.0282 (8)	0.0361 (8)	0.0392 (8)	−0.0051 (7)	0.0015 (7)	−0.0012 (7)
O1	0.0318 (6)	0.0405 (7)	0.0576 (7)	−0.0019 (5)	0.0097 (6)	−0.0059 (6)
O2	0.0349 (7)	0.0753 (11)	0.0862 (11)	0.0066 (7)	0.0110 (7)	−0.0292 (9)
O3	0.0303 (6)	0.0369 (6)	0.0414 (6)	−0.0006 (5)	0.0051 (5)	0.0039 (5)
O4	0.0268 (6)	0.0562 (8)	0.0483 (7)	0.0033 (5)	0.0052 (5)	0.0090 (6)
S1	0.0287 (2)	0.0389 (2)	0.0437 (2)	−0.00063 (17)	0.00326 (17)	−0.00318 (18)
S2	0.0290 (2)	0.0452 (2)	0.0422 (2)	−0.00065 (18)	0.00338 (17)	0.00589 (18)
C15	0.0550 (12)	0.0520 (12)	0.0560 (12)	0.0005 (10)	−0.0092 (9)	0.0086 (9)
C16	0.0598 (14)	0.0721 (16)	0.0610 (13)	0.0092 (12)	−0.0150 (11)	0.0087 (11)
C17	0.0726 (16)	0.0638 (15)	0.0585 (13)	0.0257 (12)	−0.0014 (11)	0.0158 (11)
C18	0.0692 (14)	0.0450 (11)	0.0594 (12)	0.0142 (10)	0.0079 (10)	0.0108 (9)
C19	0.0429 (10)	0.0420 (10)	0.0453 (9)	0.0038 (8)	0.0128 (8)	0.0075 (8)
C20	0.0432 (10)	0.0435 (10)	0.0470 (10)	−0.0060 (8)	0.0150 (8)	0.0060 (8)
C21	0.0758 (16)	0.0470 (12)	0.0670 (14)	−0.0151 (11)	0.0128 (12)	0.0067 (10)
C22	0.0807 (18)	0.0650 (16)	0.0771 (16)	−0.0348 (14)	0.0130 (14)	−0.0062 (13)
C23	0.0585 (14)	0.0775 (17)	0.0652 (14)	−0.0287 (13)	0.0014 (11)	−0.0046 (12)
C24	0.0410 (11)	0.0672 (14)	0.0604 (12)	−0.0145 (10)	−0.0024 (9)	0.0069 (10)
N1	0.0360 (8)	0.0433 (8)	0.0444 (8)	0.0004 (7)	0.0023 (6)	0.0083 (7)
N2	0.0338 (8)	0.0477 (9)	0.0487 (8)	−0.0080 (7)	0.0068 (6)	0.0061 (7)
O5	0.0286 (7)	0.0674 (9)	0.0516 (8)	0.0012 (6)	0.0054 (5)	0.0055 (7)

### Geometric parameters (Å, °)

**Table:**

Ni1—O1	2.0290 (13)	C12—H12	0.9300
Ni1—N1	2.0385 (15)	C13—H13	0.9300
Ni1—O5	2.0481 (14)	C14—O3	1.256 (2)
Ni1—N2	2.0682 (16)	C14—O4	1.279 (2)
Ni1—O3^i^	2.0999 (13)	C14—Ni1^i^	2.4734 (18)
Ni1—O4^i^	2.1869 (13)	O3—Ni1^i^	2.0999 (13)
Ni1—C14^i^	2.4733 (18)	O4—Ni1^i^	2.1870 (13)
C1—C6	1.402 (3)	S1—S2	2.0596 (8)
C1—C2	1.407 (2)	C15—N1	1.335 (2)
C1—C7	1.506 (2)	C15—C16	1.381 (3)
C2—C3	1.398 (2)	C15—H15	0.9300
C2—S1	1.8029 (18)	C16—C17	1.372 (3)
C3—C4	1.387 (3)	C16—H16	0.9300
C3—H3	0.9300	C17—C18	1.379 (3)
C4—C5	1.375 (3)	C17—H17	0.9300
C4—H4	0.9300	C18—C19	1.385 (3)
C5—C6	1.375 (3)	C18—H18	0.9300
C5—H5	0.9300	C19—N1	1.356 (2)
C6—H6	0.9300	C19—C20	1.483 (3)
C7—O2	1.238 (2)	C20—N2	1.345 (2)
C7—O1	1.265 (2)	C20—C21	1.395 (3)
C8—C13	1.399 (2)	C21—C22	1.383 (3)
C8—C9	1.410 (2)	C21—H21	0.9300
C8—C14	1.493 (2)	C22—C23	1.361 (4)
C9—C10	1.396 (2)	C22—H22	0.9300
C9—S2	1.7932 (17)	C23—C24	1.378 (3)
C10—C11	1.385 (3)	C23—H23	0.9300
C10—H10	0.9300	C24—N2	1.343 (2)
C11—C12	1.375 (3)	C24—H24	0.9300
C11—H11	0.9300	O5—H5A	0.841 (9)
C12—C13	1.383 (3)	O5—H5B	0.850 (9)
			
O1—Ni1—N1	93.79 (6)	C11—C12—H12	120.3
O1—Ni1—O5	90.21 (6)	C13—C12—H12	120.3
N1—Ni1—O5	99.03 (6)	C12—C13—C8	121.29 (19)
O1—Ni1—N2	173.06 (6)	C12—C13—H13	119.4
N1—Ni1—N2	79.70 (6)	C8—C13—H13	119.4
O5—Ni1—N2	93.16 (6)	O3—C14—O4	119.43 (15)
O1—Ni1—O3^i^	86.85 (5)	O3—C14—C8	119.62 (15)
N1—Ni1—O3^i^	95.52 (6)	O4—C14—C8	120.92 (15)
O5—Ni1—O3^i^	165.32 (5)	O3—C14—Ni1^i^	58.08 (9)
N2—Ni1—O3^i^	91.37 (5)	O4—C14—Ni1^i^	62.00 (9)
O1—Ni1—O4^i^	88.47 (5)	C8—C14—Ni1^i^	170.27 (12)
N1—Ni1—O4^i^	156.68 (6)	C7—O1—Ni1	132.52 (12)
O5—Ni1—O4^i^	104.18 (5)	C14—O3—Ni1^i^	91.41 (10)
N2—Ni1—O4^i^	96.58 (6)	C14—O4—Ni1^i^	86.90 (10)
O3^i^—Ni1—O4^i^	61.39 (5)	C2—S1—S2	104.94 (6)
O1—Ni1—C14^i^	84.52 (5)	C9—S2—S1	105.08 (6)
N1—Ni1—C14^i^	126.01 (6)	N1—C15—C16	122.5 (2)
O5—Ni1—C14^i^	134.86 (6)	N1—C15—H15	118.7
N2—Ni1—C14^i^	97.33 (6)	C16—C15—H15	118.7
O3^i^—Ni1—C14^i^	30.51 (5)	C17—C16—C15	118.6 (2)
O4^i^—Ni1—C14^i^	31.10 (5)	C17—C16—H16	120.7
C6—C1—C2	119.01 (17)	C15—C16—H16	120.7
C6—C1—C7	117.97 (17)	C16—C17—C18	119.46 (19)
C2—C1—C7	122.97 (15)	C16—C17—H17	120.3
C3—C2—C1	118.77 (17)	C18—C17—H17	120.3
C3—C2—S1	122.05 (15)	C17—C18—C19	119.6 (2)
C1—C2—S1	119.15 (13)	C17—C18—H18	120.2
C4—C3—C2	120.6 (2)	C19—C18—H18	120.2
C4—C3—H3	119.7	N1—C19—C18	120.67 (19)
C2—C3—H3	119.7	N1—C19—C20	115.09 (16)
C5—C4—C3	120.7 (2)	C18—C19—C20	124.23 (19)
C5—C4—H4	119.6	N2—C20—C21	121.00 (19)
C3—C4—H4	119.6	N2—C20—C19	115.26 (16)
C4—C5—C6	119.4 (2)	C21—C20—C19	123.75 (19)
C4—C5—H5	120.3	C22—C21—C20	118.9 (2)
C6—C5—H5	120.3	C22—C21—H21	120.5
C5—C6—C1	121.4 (2)	C20—C21—H21	120.5
C5—C6—H6	119.3	C23—C22—C21	119.8 (2)
C1—C6—H6	119.3	C23—C22—H22	120.1
O2—C7—O1	124.88 (17)	C21—C22—H22	120.1
O2—C7—C1	119.81 (17)	C22—C23—C24	118.7 (2)
O1—C7—C1	115.29 (15)	C22—C23—H23	120.7
C13—C8—C9	119.23 (16)	C24—C23—H23	120.7
C13—C8—C14	118.52 (16)	N2—C24—C23	122.7 (2)
C9—C8—C14	122.18 (15)	N2—C24—H24	118.6
C10—C9—C8	118.46 (16)	C23—C24—H24	118.6
C10—C9—S2	121.23 (14)	C15—N1—C19	119.12 (16)
C8—C9—S2	120.27 (13)	C15—N1—Ni1	125.65 (14)
C11—C10—C9	121.06 (18)	C19—N1—Ni1	114.83 (12)
C11—C10—H10	119.5	C24—N2—C20	118.79 (18)
C9—C10—H10	119.5	C24—N2—Ni1	126.75 (14)
C12—C11—C10	120.63 (19)	C20—N2—Ni1	114.19 (12)
C12—C11—H11	119.7	Ni1—O5—H5A	108.9 (17)
C10—C11—H11	119.7	Ni1—O5—H5B	102.6 (18)
C11—C12—C13	119.32 (19)	H5A—O5—H5B	110.3 (14)

Symmetry codes: (i) −*x*+1, −*y*, −*z*+2.

### Hydrogen-bond geometry (Å, °)

**Table:**

*D*—H···*A*	*D*—H	H···*A*	*D*···*A*	*D*—H···*A*
O5—H5B···O2	0.85 (1)	1.82 (1)	2.646 (2)	162 (2)
O5—H5A···O4^ii^	0.84 (1)	1.89 (1)	2.7187 (18)	169 (2)

Symmetry codes: (ii) *x*+1, *y*, *z*.
